# Incidence and risk factors for food hypersensitivity in UK infants: results from a birth cohort study

**DOI:** 10.1186/s13601-016-0089-8

**Published:** 2016-01-26

**Authors:** Kate E. C. Grimshaw, Trevor Bryant, Erin M. Oliver, Jane Martin, Joe Maskell, Terri Kemp, E. N. Clare Mills, Keith D. Foote, Barrie M. Margetts, Kirsten Beyer, Graham Roberts

**Affiliations:** 1 Clinical and Experimental Sciences and Human Development in Health Academic Unit, Faculty of Medicine, University of Southampton, Mailpoint 803, Level F, South Academic Block, Southampton, SO16 6YD UK; 2 Department of Nutrition and Dietetics, Southampton Children’s Hospital, Southampton, SO16 6YD UK; 3 Primary Care and Population Sciences Academic Unit, Faculty of Medicine, University of Southampton, Southampton, SO16 6YD UK; 4 NIHR Clinical Research Facility, University Hospital Southampton NHS Foundation Trust, Southampton, SO16 6YD UK; 5 The Hampshire Hospitals Foundation Trust, Winchester, SO22 5DG UK; 6 Institute of Inflammation and Repair, Manchester Academic Health Science Centre, Manchester Institute of Biotechnology, The University of Manchester, Manchester, M1 7DN UK; 7 Department of Paediatric Pneumology and Immunology, Charité University Medical Centre, Berlin, Germany; 8 NIHR Respiratory Biomedical Research Unit, University Hospital Southampton NHS Foundation Trust, Southampton, SO16 6YD UK

**Keywords:** Food hypersensitivity, Food allergy, Epidemiology, EuroPrevall, Incidence, Risk factors, Dietary pattern analysis, Healthy eating

## Abstract

**Background:**

The prevalence of food hypersensitivity in the UK is still largely open to debate. Additionally its pathogenesis is also unclear although it is known that there are differing phenotypes. Determining its prevalence, along with identifying those factors associated with its development will help to assess its clinical importance within the national setting and also add to the debate on appropriate prevention strategies.

**Methods:**

A population based birth cohort study conducted in Hampshire, UK as part of the EuroPrevall birth cohort study. 1140 infants were recruited with 823 being followed up until 2 years of age. Infants with suspected food reactions were assessed including specific IgE measurement and skin prick testing. Diagnosis of food hypersensitivity was by positive double-blind, placebo-controlled food challenge (DBPCFC) where symptoms up to 48 h after the end of the food challenge were considered indicative of a food hypersensitivity. Factors associated with food hypersensitivity and its two phenotypes of IgE-mediated and non-IgE-mediated disease were modelled in a multivariable logistic regression analysis.

**Results:**

Cumulative incidence of food hypersensitivity by 2 years of age was 5.0 %. The cumulative incidence for individual food allergens were hens’ egg 2.7 % (1.6–3.8); cows’ milk 2.4 % (1.4–3.5); peanut 0.7 % (0.1–1.3); soy 0.4 % (0.0–0.8); wheat 0.2 % (0.0–0.5) and 0.1 % (0.0–0.32) for fish. The cumulative incidence of IgE-mediated food allergy was 2.6 % with 2.1 % reacting to hens’ egg. For non-IgE-mediated food allergy the cumulative incidence was 2.4 % (cows’ milk 1.7 %). Predictors for any food hypersensitivity were wheeze, maternal atopy, increasing gestational age, age at first solid food introduction and mean healthy dietary pattern score. Predictors for IgE mediated allergy were eczema, rhinitis and healthy dietary pattern score whereas for non-IgE-mediated food allergy the predictors were dog in the home, healthy dietary pattern score, maternal consumption of probiotics during breastfeeding and age at first solid food introduction.

**Conclusions:**

Just under half the infants with confirmed food hypersensitivity had no demonstrable IgE. In an exploratory analysis, risk factors for this phenotype of food hypersensitivity differed from those for IgE-mediated food allergy except for a healthy infant diet which was associated with less risk for both phenotypes.

**Electronic supplementary material:**

The online version of this article (doi:10.1186/s13601-016-0089-8) contains supplementary material, which is available to authorized users.

## Background

Food hypersensitivity continues to be a concern for health care professionals and the general population since it adversely affects quality of life [1, 2] and household and healthcare expenditure [3, 4]. Any public health concern needs local incidence and prevalence data [5] and this is particularly relevant for food hypersensitivity due to conflicting opinions as to prevalence [6, 7]. There is also great interest in its potential risk factors due to its association with the development of other conditions [8, 9]. Since geographical issues are also important in the development of food hypersensitivity, not all previously identified risk factors may be associated with food hypersensitivity development in a UK cohort in infants. One study has looked at risk factors of food hypersensitivity development in the UK but it only considered dietary factors [10]. We have previously described the relationship between breast feeding, complementary feeding and dietary patterns and food hypersensitivity in this cohort. In this publication we now present data on the incidence of food allergy in the UK within a general UK cohort and describes the results of a follow up of 1140 infants pairs. In an additional exploratory analysis, we also aimed to investigate the possible risk factors for IgE and non-IgE mediated food allergy.

## Methods

### Study design

The PIFA (prevalence of infant food allergy) study is the UK cohort of the EuroPrevall project [11] which recruited 1140 babies between 2006 and 2008. Its design has been described elsewhere [11, 12] but the main points of its methodology are reported here. It received approval from Research and Development departments at the Royal Hampshire County Hospital, Winchester and Southampton General Hospital and ethical approval was granted by North and Mid Hampshire Local Research Ethics Committee and Southampton and South West Hampshire Local Research Ethics Committee (05/Q1703/34).

**Fig. 1:**
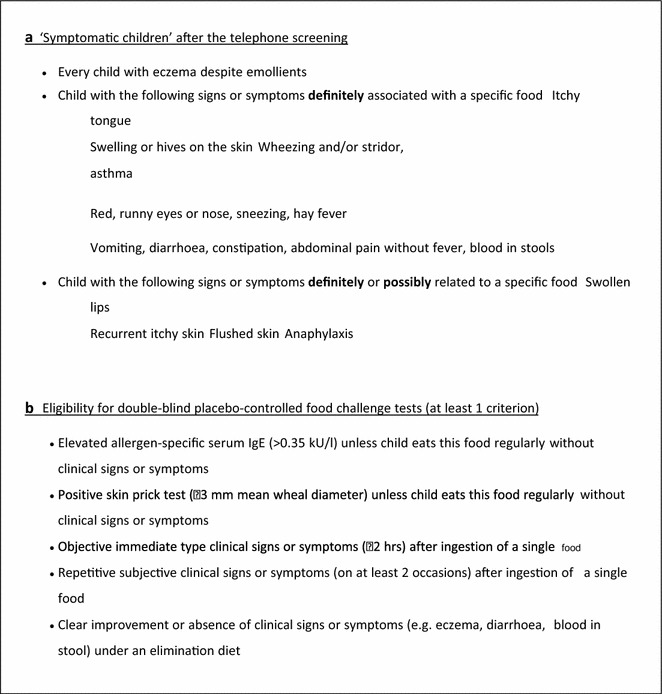
Criteria for **a** defining ‘symptomatic children’ after telephone screening; **b** eligibility for double-blind placebo-controlled food challenge tests (used with Permission from Keil et al. [12])

All eligible pregnant women registered with the Hampshire Hospitals Foundation Trust midwifery service were invited to take part in the study. Interested women met one of the study research fellows when informed consent was taken and baseline information on socio-economic, environmental and family allergy history was collected [12]. Potential reactions to food were identified via parental reporting and the 12 and 24 month questionnaires. Those infants displaying signs or symptoms which fulfilling the EuroPrevall-wide criteria for assessment as described in Keil et al. [12] and also detailed in Fig. 1 were invited to attend the Southampton Wellcome Trust Clinical Research Facility (WTCRF). Those who went on to meet the eligibility criteria to perform a double-blind, placebo-controlled challenge (DBPCFC) of an (Fig. 1) returned on two subsequent days for this to be carried out. Additional dietary intake data was invited from all study participants in the form of a prospective food diary kept for the first year of life [13, 14]. The study received ethics approval as detailed previously [13, 14].

### Food hypersensitivity definitions

‘Parent perceived food hypersensitivity’ describes when a parent suspected their child had a reaction to food. A positive double-blind, placebo-controlled challenge (DBPCFC) was defined as either (1) objective immediate type symptoms or signs within 2 h of the final dose and/or (2) parentally reported objective signs of delayed reactions (vomiting, diarrhoea, hives and worsening of eczema), up to 48 h after challenge. With a positive DBPCFC, if there was a positive specific IgE (SpIgE) (≥0.35 kU_l_/L) or skin prick test (SPT) (wheal diameter ≥3 mm), the infant was described as food allergic [15]. Food hypersensitivity without sensitisation was described as ‘non-IgE food hypersensitivity’. ‘Food hypersensitivity’ is used to describe all positive DBPCFC.

### Control infants

Every child with DBPCFC diagnosed food hypersensitivity was allocated two age-matched controls, who were selected by approaching parents of infants with birthdays just before or after the child with a confirmed food hypersensitivity until two controls were found. Since the control child was not selected until after a positive food challenge, some months may have passed between the time of the initial assessment of the case and the initial assessment of the control. There was however, never more than 4 weeks between the positive food challenge result and the assessment of the control child. Each control child was assessed in the same manner (apart from SPT) as the symptomatic infants, including a blood sample, to ensure they exhibited no signs of food allergy [12].

### Statistical analysis

Analysis was carried out using SPSS version 21 (IBM, New York, USA) and STATA version 12 (College Station, USA). The infant healthy eating pattern score was derived by PCA analysis of the food diet data as described previously and describes the dietary component which accounted for 50 % of the variance and had high positive values associated with fruit, vegetable, fish and poultry consumption. Low/negative values associated with highly processed adult foods (such as ready meals, cook in-sauces, potato products and bacon) and the use of commercial baby foods more than once a day [13]. Cumulative incidence of food hypersensitivity was calculated with 95 % confidence intervals. Exploratory analyses assessed whether any potentially important exposures were significantly related to food hypersensitivity. A univariate logistic regression approach was taken followed by backwards multivariable analysis (which initially included factors with p < 0.1). Where the cells formed by the outcome and categorical predictor variables had no observations, exact logistic regression or the Bayesian firth logic approach was used to calculate odds ratios [16]. As an example of power for this exploratory analysis, with exposure rates of 62 and 35 % in cases and controls respectively and 41 cases and 82 controls, we had 80 % power to detect a significant relationship between the exposure and outcome at a 5 % level of significance (STATA version 12).

## Results

### Participants

**Fig. 2:**
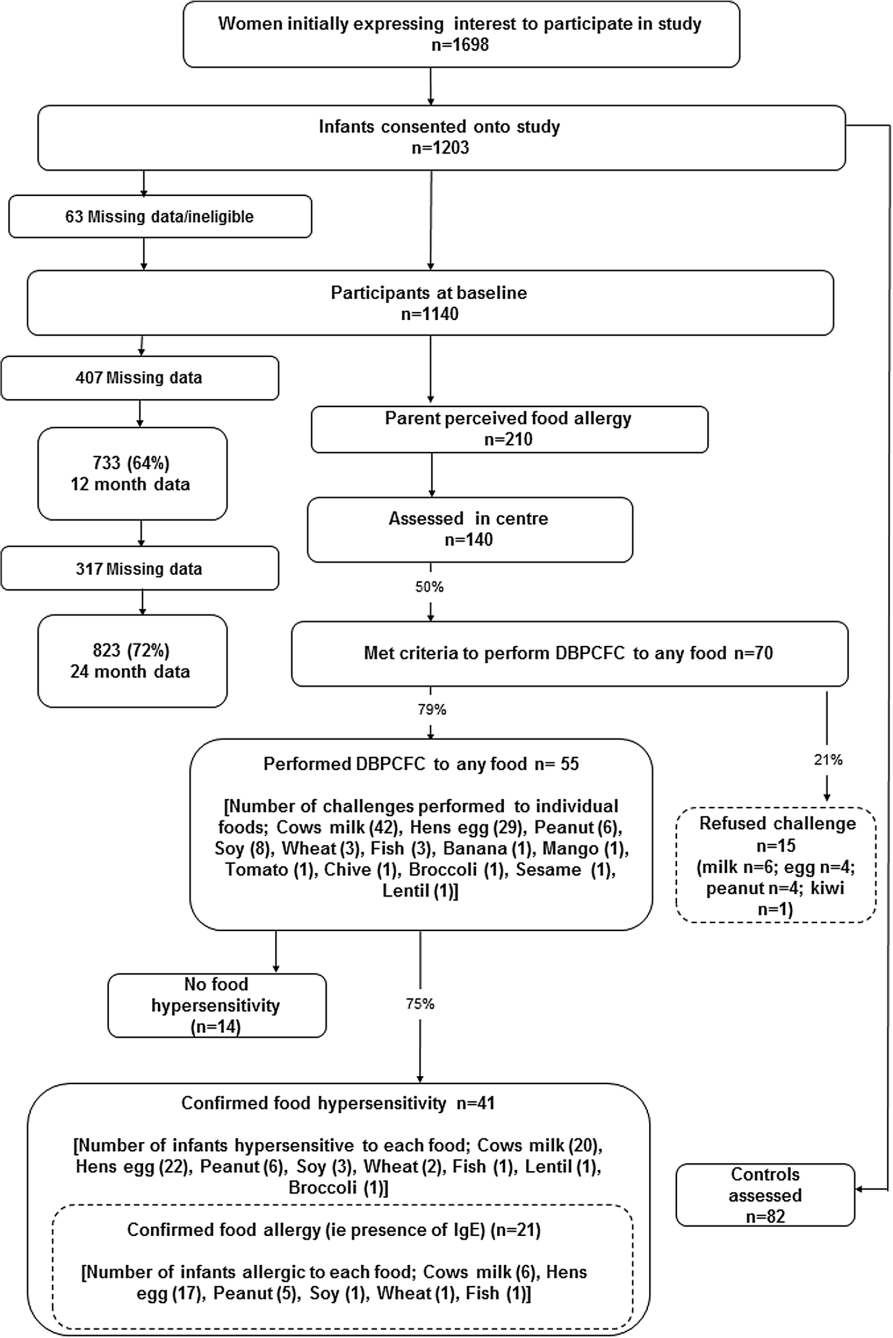
Flow of participants through the study

1605 pregnant women initially expressed an interest in participating in the study. 402 mothers subsequently declined, mainly due to time commitments or unwillingness for their child to have blood tests. We had no ethical approval to collect further data about these women. 1203 infants were born of which 63 were excluded either because they did not satisfy the inclusion criteria (due to low apgar, premature delivery or delivery outside the recruitment period) or they had missing birth data. The resultant baseline cohort was 1140 (Table 1). A total of 823 (72.2 %) infants were followed up to 2 years (Fig. 2).

**Table 1:** Demographic, socioeconomic and familial factors of the study participants

	All participants	Lost to follow up (2 years)	Eligible for DBPCFC		
			Not challenged	Challenged	Confirmed food hypersensitivity
n	1140	317	15	55	41
Baseline characteristics					
Caucasian ethnicity	1093 (95.8 %)	304 (96.0 %)	11 (73.3 %)	50 (90.9 %)	39 (95.1 %)
Smoking during pregnancy	70 (6.1 %)	45 (14.2 %)	0 (0.0 %)	1 (1.8 %)	1 (2.4 %)
Mothers’ mean age, years	31.9 (5.2)	30.0 (5.8)	31.9 (5.2)	32.0 (5.5)	31.6 (5.3)
Fathers’ mean age, years	34.1 (5.7)	32.6 (6.4)	33.9 (5.1)	33.9 (4.5)	33.2 (4.6)
Highest education of parents					
Low (up to 12y)	220 (19.3 %)	86 (27.2 %)	4 (26.7 %)	9 (16.4 %)	7 (17.1 %)
Intermediate (>12 years, e.g. college)	331 (29.0 %)	96 (30.2 %)	3 (20.0 %)	19 (34.5 %)	13 (31.7 %)
High (e.g. university)	565 (49.6 %)	121 (38.3 %)	8 (53.3 %)	27 (49.1 %)	21 (51.2 %)
Allergies in family					
Maternal atopy (A, AR or E)	765 (67.1 %)	210 (66.4 %)	13 (86.7 %)	48 (87.3 %)	36 (87.8 %)
Paternal atopy (A, AR or E)	610 (53.5 %)	170 (53.7 %)	11 (73.3 %)	30 (54.5 %)	23 (56.1 %)
Maternal food hypersensitivity	249 (21.8 %)	77 (24.4 %)	6 (40.0 %)	13 (23.6 %)	11 (26.8 %)
Paternal food hypersensitivity	135 (11.8 %)	35 (11.1 %)	1 (6.7 %)	7 (12.7 %)	6 (14.6 %)
Urban living environment	273 (23.9 %)	99 (31.2 %)	6 (40.0 %)	11 (20.0 %)	8 (19.5 %)
Mean number of sibs at home	1.7 (0.88)	1.8 (0.99)	1.4 (0.65)	1.6 (0.71)	1.5 (0.64)
Female sex	557 (48.9 %)	166 (52.4 %)	6 (40.0 %)	23 (41.8 %)	17 (41.5 %)
Animals in household at birth					
Any	566 (49.6 %)	164 (50.6 %)	8 (53.3 %)	33 (60.0 %)	26 (63.4 %)
Cat	325 (28.5 %)	92 (28.4 %)	4 (26.6 %)	15 (27.3 %)	10 (24.4 %)
Dog	204 (17.9 %)	66 (20.4 %)	3 (20.0 %)	15 (27.3 %)	13 (31.7 %)
Season of birth (n = 1139)					
Summer	379 (33.3)	112 (34.6 %)	2 (13.3 %)	21 (38.2 %)	17 (41.5 %)
Autumn	251 (22.0)	66 (20.4 %)	7 (46.6 %)	13 (23.6 %)	8 (19.5 %)
Winter	183 (16.1)	51 (15.7)	2 (13.3 %)	9 (16.4 %)	8 (19.5 %)
Spring	326 (28.6)	95 (29.3 %)	4 (26.6 %)	11 (20.0 %)	8 (19.5 %)

Figures represent numbers (%) or mean (SD)

*A* asthma, *AR* allergic rhinitis, *E* eczema

### Cumulative incidence of food hypersensitivity

210 infants (25.5 %; 95 % CI 22.5–28.5) had parental perceived food hypersensitivity. 173 of these were identified via parental phone call to the study office and 61 were identified via administration of the questionnaire (55 at 12 months and 10 at 24 months). Of these 210 infants, 135 met the criteria for assessment and were invited for clinical assessment. Those not invited for assessment had other presentations, e.g. lactose intolerance and perioral rash with acidic foods. Of the 135 infants who underwent clinical assessment, 70 were eligible for a DBPCFC. Fifty-five infants underwent the DBPCFC and 41 of these had a positive DBPCFC giving a cumulative incidence of food hypersensitivity of 5.0 % (95 % CI 3.5–6.5). The cumulative incidence for individual foods were hens’ egg 2.7 % (1.6–3.8); cows’ milk 2.4 % (1.4–3.5); peanut 0.7 % (0.1–1.3); soy 0.4 % (0.0–0.8); wheat 0.2 % (0.0–0.5) and 0.1 % (0.0–0.32) for fish, lentil and broccoli (Fig. 1). Twelve infants were reactive to more than one food. The commonest parentally reported symptoms at assessment were physician diagnosed eczema (12 infants) and vomiting (11 infants).

### Characteristics of participants with food hypersensitivity

Of the 41 infants with DBPCFC confirmed food hypersensitivity, 38 had SpIgE assessed and 40 were skin prick tested. Eczema was the presenting symptoms in 16 (39.0 %) of children with food hypersensitivity; gastrointestinal symptoms were the next most frequent (26.8 %). No child presented with a history of anaphylaxis (Table 2).

**Table 2:** Characteristics of participants with food hypersensitivity and their controls at initial assessment

	Participants with food hypersensitivity (n = 41)	Control participants (n = 82)	p value
Median (IQR) age of child, months	9.3 (5.7–17.0)	14.9 (10.1–20.2)	0.024
Diagnostic criteria: positive DBPCFC	41 (100 %)		
History of anaphylaxis	0 (0 %)		
Presenting symptoms			
Gastrointestinal	11 (26.8 %)		
Cutaneous (eczema and/or urticaria)	22 (46.3 %)		
Respiratory	4 (9.7 %)		
Other	3 (7.3 %)		
None	1 (2.4 %)		
How soon did symptoms appear, minutes (SE) [Range]	170 (118) [0–4320]		
Positive specific IgE (≥0.35 kU/l)	21 (51.2 %)		
Median specific IgE, kU/l	1.85 (0.85–3.81)		
Positive SPT (≥3 mm)	17 (41.4 %)		
Median SPT weal diameter, mm	5.00 (3.00–6.25)		
Eczema	32 (78.1 %)	45 (54.9 %)	0.005
Wheeze	17 (41.5 %)	21 (25.6 %)	0.054
Rhinitis	14 (34.1 %)	11 (13.4 %)	0.007

Assessment refers to the assessment of cases at presentation with food hypersensitivity and the equivalent assessment of their controls. Figures represent numbers (%) or mean (SD) or median (IQR)

Specific IgE and SPT data refer to the specific food that is being assessed in individual participants, where there is more than one food, the highest result is taken as representative. One case had no presenting symptoms as they were found to have a positive specific IgE to peanut on assessment as a control participant. P values represent a comparison between case of food hypersensitivity and controls; Chi squared test for categorical data, Mann–Whitney U test for non-parametric data or a two-sample t test for parametric data

*DBPCFC* double-blind, placebo-controlled food challenge

### Potential risk factors for food hypersensitivity

Paternal, perinatal, environmental and infant nutritional factors were compared between cases of food hypersensitivity and controls (Tables 2, 3, 4; Additional file 1: Table S1). Data relating infant diet to food hypersensitivity has been published in detail elsewhere [14, 15].

**Table 3:** Maternal, parental, perinatal, environmental health and medicinal factors

	Participants with food hypersensitivity (n = 41)	Control participants (n = 82)	p value
Maternal atopy	36 (87.8 %)	52 (63.4 %)	0.002
Paternal atopy	23 (57.5 %)	50 (61.0 %)	0.844
Mean maternal pre-pregnancy weight, kg	65.9 (14.0)	64.0 (11.5)	0.558
Mean maternal pre-pregnancy height, cm	164.1 (5.9)	165.0 (6.6)	0.690
Median parity	0.0 (0.0–1.0)	0.0 (0.0–1.0)	0.729
Singleton pregnancy	40 (97.6 %)	82 (100.0 %)	0.333
Maternal smoking in pregnancy	1 (2.4 %)	3 (3.7 %)	1.000
Other household smoking in pregnancy	8 (19.5 %)	5 (6.1 %)	0.027
Aspirin/paracetamol during pregnancy	30 (81.1 %)	49 (69.0 %)	0.132
Any anti-inflammatory during pregnancy	4 (11.1 %)	7 (9.9 %)	0.542
Maternal antibiotics during pregnancy	9 (22.0 %)	19 (23.3 %)	0.286
Mode of delivery			
Normal	20 (51.3 %)	37 (45.7 %)	0.860
Caesarean	13 (31.7 %)	20 (24.4 %)	
Forceps	3 (7.3 %)	12 (14.8 %)	
Mean gestation, weeks	39.5 (1.7)	40.0 (1.4)	0.062
Mean birth weight, grams	3480 (470)	3370 (562)	0.913
Antibiotics in first week of age	0 (0.0 %)	0 (0.0 %)	1.000
Environmental			
Urban living Environment	8 (19.5 %)	11 (13.4 %)	0.601
Live on a main road	3 (7.3 %)	5 (6.1 %)	0.535
Cat at home	10 (24.4 %)	24 (29.3 %)	0.365
Dog at home	13 (31.7 %)	12 (14.6 %)	0.026
Mould in home	5 (12.2 %)	8 (9.8 %)	0.457
Type of flooring where baby sleeps			
Carpet	37 (90.2 %)	72 (87.8 %)	0.757
Wooden, laminate or parquet	4 (9.8 %)	9 (11.0 %)	
Linoleum or vinyl tiles	0 (0.0 %)	1 (1.2 %)	
Type of mattress your baby sleeps on			
Foam	29 (70.7 %)	51 (62.2 %)	0.362
Synthetic	10 (24.4 %)	22 (26.8 %)	
Other	2 (4.9 %)	9 (11.0 %)	
Cleaning kitchen work surfaces			
Non-bactericidal	12 (29.3 %)	27 (32.9 %)	0.396
Bactericidal	27 (65.9 %)	50 (61.0 %)	
Neither	1 (2.4 %)	5 (6.1 %)	
Do not know	1 (2.4 %)	0 (0.0 %)	
Cleaning table where you eat			
Spray cleaner	26 (63.4 %)	38 (46.3 %)	0.131
Soap and Water	10 (24.4 %)	19 (23.2 %)	
Just water	2 (4.9 %)	6 (7.3 %)	
None of these	3 (7.3 %)	19 (23.2 %)	
Pacifier/dummy			
Latex	5 (12.2 %)	8 (9.8 %)	0.202
Silicon	21 (51.2 %)	32 (39.0 %)	
Attendance at day care or a nursery	13 (31.7 %)	19 (23.2 %)	0.246
Mean age when started day care or a nursery, months	7.3 (2.19)	8.74 (3.74)	0.269
Health			
Upper respiratory infection			
None	9 (23.7 %)	21 (27.6 %)	0.856
Occasionally	20 (52.6 %)	36 (47.4 %)	
Often	9 (23.7 %)	19 (25.0 %)	
Lower respiratory infection			
None	34 (89.5 %)	71 (93.4 %)	0.462
Occasionally	4 (10.5 %)	4 (5.3 %)	
Often	0 (0.0 %)	1 (1.3 %)	
Wheeze with upper respiratory infection			
None	24 (63.2 %)	59 (77.6 %)	0.195
Occasionally	11 (28.9 %)	15 (19.7 %)	
Often	3 (7.9 %)	2 (2.6 %)	
Bronchiolitis (bronchitis)			
None	36 (94.7 %)	75 (98.7 %)	0.106
Occasionally	2 (5.3 %)	1 (1.3 %)	
Often	0 (0.0 %)	0 (0.0 %)	
Middle ear infection			
None	32 (84.2 %)	70 (92.1 %)	0.165
Occasionally	6 (15.8 %)	6 (7.9 %)	
Often	0 (0.0 %)	0 (0 %)	
Gastrointestinal illness			
None	30 (78.9 %)	69 (90.8 %)	0.125
Occasionally	7 (18.4 %)	7 (9.2 %)	
Often	1 (2.6 %)	0 (0.0 %)	
Medication			
Median number of antibiotics in last 12 months	1 (0–2)	0.5 (0–1)	0.341
Mean age when first received antibiotics, months	8.65 (7.04)	8.44 (6.59)	0.964
Received aspirin	1 (2.6 %)	0 (0.0 %)	0.333
Received paracetamol	38 (100.0 %)	74 (97.4 %)	0.442
Received anti-inflammatories (e.g. Ibuprofen, Nurofen)	29 (76.3 %)	51 (67.1 %)	0.214
Received anti-reflux medication	11 (28.9 %)	10 (13.2 %)	0.039
Received any vaccinations	37 (97.4 %)	73 (96.0 %)	0.565
Received any skin creams, lotions or powders	33 (86.8 %)	55 (72.4 %)	0.079

Figures are numbers (%) in each group, means (SD) or medians (25th, 75th centiles) unless specified. P values relate to a comparison between cases and control; they represent a Chi squared test for categorical data, Mann–Whitney U test for non-parametric data and two sample t test for parametric data. All data was not available for all participants. Maternal atopy defined as any of maternal asthma, eczema, rhinitis or food allergy

**Table 4:** Univariate and multivariable analyses for infants with food hypersensitivity, IgE-mediated food allergy and non-IgE-mediated hypersensitivity

	All infants will food hypersensitivity (n = 41) compared to control infants			
	Univariate		Multivariable*	
	Odds ratio (95 % CI)	p value	Odds ratio (95 % CI)	p value
Wheeze, (at initial assessment)	2.120 (0.940–4.782)	0.092	20.591 (1.465–289.341)	0.025
Maternal atopy	5.192 (1.683–16.017)	0.002	87.479 (1.021–7498.366)	0.049
Gestation, weeks	0.756 (0.585–0.978)	0.033	0.171 (0.045–0.642)	0.009
Age at first solids, weeks	0.920 (0.830–0.998)	0.044	0.506 (0.282–0.908)	0.022
Mean infant healthy eating dietary pattern score, arbitrary units	0.365 (0.229–0.583)	0.002	0.155 (0.028–0.868)	0.034

	IgE-mediated (n = 21) compared to control infants				Non-IgE-mediated (n = 20) compared to control infants			
	Univariate		Multivariable**		Univariate		Multivariable**	
	Odds ratio	p	Odds ratio	p	Odds ratio	p	Odds ratio	p
Eczema (at initial assessment)	17.83* (2.89–∞)	<0.001	18.67**** (1.03–338.41)	0.048				
Rhinitis (at initial assessment)	3.94 (1.31–11.83)	0.023	4.80**** (1.19–19.36)	0.027	2.96 (0.92–9.52)	0.087		
Maternal atopy	11.54 (1.47–90.35)	0.003						
Vitamin D supplement during pregnancy	8.68*** (0.66–∞)	0.097						
Age at first egg from any source, months	1.05 (1.00–1.11)	0.026	Not included in analysis as likely reverse causality					
Dog in the home	3.24 (1.00–10.48)	0.076			4.37 (1.38–13.80)	0.015	19.49 (1.17–325.93)	0.039
Wheeze associated with upper respiratory tract	2.84 (1.01–7.98)	0.052						
Healthy eating dietary pattern score, arbitrary units	0.36 (0.20–0.66)	0.001	0.32**** (0.16–0.66)	0.012	0.34 (0.19–0.62)	<0.001	0.28 (0.09–0.87)	0.028
Maternal age, years					0.87 (0.78–0.99)	0.037		
Paternal age, years					0.893 (0.803–0.992)	0.035		
Maternal food hypersensitivity					2.790 (0.994–7.831)	0.055		
Other household smoking					5.133 (1.32–19.95)	0.023		
Consumed probiotics whilst breastfeeding					3.31 (1.13–9.75)	0.084	45.41 (3.41–604.67)	0.004
Age at first solid, months					0.84 (0.73–0.97)	0.021	0.60 (0.40–0.89)	0.011
Milk overlap, months					0.94 (0.88–1.00)	0.037		
Received anti-reflux medication					3.312 (1.13–9.75)	0.030	Not included in analysis as likely reverse causality	

Adjusted and unadjusted odds ratios (95 % confidence intervals (CI) and p values) are presented for all factors significant in the multivariate model

* Factors associated with hypersensitivity at a p value <0.1 were entered into a multivariable analysis using SPSS. A stepwise backwards selection process was used

** For multivariable analysis p values are only given for those variables included in the final model

*** Exact logistic regression model used to estimate parameters

**** Firthlogit approach to fit a logistic model by penalized maximum likelihood regression (accessed via STATA)

Factors significantly associated with food hypersensitivity were eczema, rhinitis, maternal atopy, anti-reflux medication, other household smoking, dog in the home and a variety of dietary factors including age at first solids, infant healthy eating dietary pattern score and reduced intake of milk whilst breastfeeding. (Tables 2, 3; Additional file 1: Table S1). The ‘infant received anti-reflux medication’ and ‘reduced maternal milk intake whilst breastfeeding’ variables were removed from further analyses since any association was likely to be due to reverse causality. The variable ‘other household smoking’ was also removed from further analyses since the significant association seen may be due to the low level seen in the control infants compared to all study participants and not a causal relationship. All the factors related to food hypersensitivity (p < 0.1) were included in the multivariable analysis (see Tables 2, 3; Additional file 1: Table S1–S9). After the multivariable analysis wheeze, maternal atopy, gestational age, age at first solids and mean healthy eating dietary pattern score remained independent factors in the model. Wheeze (aOR 20.59) and maternal atopy (aOR 87.48) were factors that increased risk of food hypersensitivity. Increasing gestational age (aOR 0.171), age at solid introduction (aOR 0.506) and healthy eating dietary pattern score (aOR 0.155) were associated with a significant reduction in risk (Table 4).

### IgE-mediated food allergy compared to non-IgE-mediated food hypersensitivity

21 infants (cumulative incidence 2.6 %, 1.52–3.6) had IgE-mediated food allergy. Incidences for individuals foods were hens’ egg 2.1 % (1.1–3.0); cows’ milk 0.7 % (0.2–1.3); peanut 0.6 % (0.1–1.1); and 0.1 % (0.0–0.4) for soy, wheat and fish. Eight infants reacted to more than one food. Infants with non-IgE-mediated food hypersensitivity reacted most frequently to cows’ milk (1.7 %, 0.8–2.6) then hens’ egg (0.6 %, 0.1–1.1; soy (0.2 %, 0.0–0.6); and peanut, wheat, lentil and broccoli (0.1 %, 0.0–0.4). Four infants reacted to more than one food. No infant had both IgE-mediated allergy and non-IgE-mediated food reactions (Additional file 1: Table S2) and no infant with IgE ≥0.35 kU/l had symptoms after 2 h of ingesting the culprit food (Additional file 1: Table S3).

There were significant differences between the two phenotypes of food hypersensitivity. When comparing the two phenotypes, significant differences were found for maternal food hypersensitivity (0.013), mean time to reaction after food ingestion (p = 0.007), current eczema (p = 0.028), an urban living environment (p = 0.015), fish oil supplement use during pregnancy (p = 0.025), probiotic use during breastfeeding (p = 0.009) and day care attendance (p = 0.025) (Additional file 1: Table S3).

Risk factors for IgE- and non-IgE-mediated reactions differed (Additional file 1: Tables S4–S9). For infants with IgE-mediated food allergy (n = 21), they were current eczema, current rhinitis, maternal atopy, wheeze with an upper respiratory tract infection, vitamin D supplementation during pregnancy and infant healthy infant diet score. In a multivariable analysis, the independent risk factors for IgE-mediated food allergy were eczema (aOR 18.67, 1.03–338.41), rhinitis (aOR 4.80, 1.19–19.36) and infant healthy eating dietary pattern score (aOR 0.32, 0.16–0.66). In contrast, for infants with non-IgE-mediated food hypersensitivity (n = 20), the risk factors were a dog in the home, infant healthy eating dietary pattern score, maternal age, paternal age, other household smoking, anti-reflux medication use, age at first solids and concurrent breastfeeding with cows’ milk from any source. In a multivariable analysis, the independent risk factors for non-IgE-mediated food hypersensitivity were dog in the home (aOR 19.49, 1.17–325.93), consuming probiotics whilst breastfeeding (aOR 45.41, 3.41–604.67), age at first solid food introduction (aOR 0.60, 0.40–0.89) and infant healthy eating dietary pattern score (aOR 0.28, 0.09–0.87) (Table 4).

## Discussion

In this UK cohort we found the cumulative incidence of DBPCFC confirmed food hypersensitivity to be 5.0 % (3.7–6.7). This is a little higher than other published figures of 0.0–4.2 % in the 0–5 year age group [5]. This is probably due to methodological differences since many studies only performed DBPCFC if there was sensitisation thus potentially missing non-IgE-mediated food hypersensitivity. The observed difference may also be due to geographical differences since it is thought the incidence of food hypersensitivity reactions is higher in Northern European countries [5].

Since all children with a clinical history of food hypersensitivity were challenged in this study, a cumulative incidence for both IgE- and non-IgE-mediated conditions could be determined: 2.6 % (1.5–3.6) for IgE-mediated food allergy and 2.4 % (1.4–3.5) for non-IgE-mediated food hypersensitivity. There is a lack of published data for non-IgE-mediated food hypersensitivity for comparison, but our results are broadly similar to those published in the most recent systematic review [17] although they are somewhat higher for hens’ egg (2.1 % compared to 0.0–1.7 %).

Novel to this study was the ability to look at risk factors for differing phenotypes of food hypersensitivity. Our exploratory analysis showed the factors associated with food hypersensitivity were wheeze, maternal atopy, gestation, age at first solids and healthy eating dietary pattern, which are similar to those found in previous research [18–25]. For IgE-mediated disease, the multivariable analysis demonstrated eczema, rhinitis and healthy eating pattern to be significant independent risk factors. The association between eczema and food allergy has long been recognised [26] and the opinion that eczema is a likely risk factor for food allergy is becoming more widely accepted due to data relating filaggrin gene defects with the development of eczema, allergic sensitization, and asthma and allergic rhinitis [27–29]. Filaggrin gene defect leads to a damaged skin barrier which increases permeability to exogenous proteins and possibly exposure of the innate immune system to allergens [30]. This phenomenon may also account for rhinitis being a risk factor for food allergy since the infant may also have become sensitised to aeroallergens through a damaged epithelial layer [31]. A healthy eating pattern was identified as being protective against the development of food allergy with the possible mechanism being the immunomodulatory effect of nutrients found in fruit and vegetables which were a feature of the observed healthy eating pattern along with a predominantly home-cooked diet [13]. Fruit and vegetables are good sources of vitamin C, beta-carotenes, folate and oligo-saccharides all of which have been shown to have immunomodulatory actions [32–34]. Also, home processed foods may have a higher microbial load than commercially prepared foods [35] and this may offer protection from the development of allergic disease as suggested by the “Hygiene Hypothesis” [36].

In the multivariable analysis for infants with non-IgE-mediated food hypersensitivity, dog in the home, healthy eating pattern, probiotic whilst breastfeeding and younger age at first solid food introduction were independent risk factors. The nature of the relationship between pet ownership and allergy development is still under debate [37–40]. If a protective effect is only seen for IgE-mediated conditions then that may explain the conflicting findings in the literature. It is recognised that pet ownership results in altered household microbial communities [41] and that these differences can lead to altered gut flora [42]. Whilst this may lead to changes in the immune system which reduce the risk of IgE-mediated allergy [43] this altered gut flora can adversely affect digestive enzyme activity, particularly of lactase. This is a previously reported phenomenon [44] and since the majority of infants with non-IgE-mediated food hypersensitivity were reactive to milk, this is a potential mechanism for these cases.

The effect of altered gut microbiota may also be the reason for other observed associations in these infants including healthy eating pattern and probiotic consumption during breastfeeding. The healthy eating dietary pattern may have a protective effect on gut health since it contained large amounts of home processed fruits and vegetables which are good sources of oligo-saccharides which, as naturally occurring prebiotics, promote gut colonization by bifidobacterium [45]. The observed association between probiotic consumption during breastfeeding and non-IgE-mediated food hypersensitivity is not readily explained but it can be hypothesised that it relates to the effect of the infant gut flora on gut enzyme activity. There is evidence that probiotics taken when breastfeeding alter breast milk composition [46] and that this may reduce the risk of allergy development in the infant [47]. However, there is no data describing the effect of maternal consumption of probiotic during lactation on the infant gut flora but it could be hypothesised that it increases the bifidobacteria colonisation that has been reported during breastfeeding [48] thus enhancing the effect of the breast milk on gut flora further. At breastfeeding cessation the infant’s gut flora becomes predominantly populated by coliform and bacteroid bacteria [48] which do not aid gut enzyme activity like hybridisable bacteria does [49] so the sudden change in the gut enzyme promoting environment may cause the observed Non-IgE mediated symptoms.

The age at first solid food introduction was significantly different for food hypersensitivity and for non-IgE-mediated food hypersensitivity. This observation could explain why some previous studies found an association between the early introduction of solids and food hypersensitivity [23, 24] and others have not [50, 51] since most studies do not identify the phenotype of hypersensitivity being investigated. It is considered that the infant gut is relatively immature before age 4–6 months and introducing solids before this time could cause food hypersensitivity symptoms, due to physiological mechanisms such as high intestinal permeability [52] which could upset the normal homeostasis of mucosal cell transport processes [53].

**Fig. 3:**
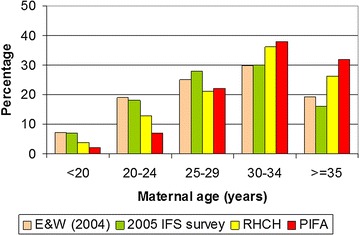
Comparison between maternal age in UK birth cohort and similar populations. Percentage of mothers in each age range at enrolment into the UK birth cohort (PIFA) in comparison with England and Wales population; 2004 (E&W), 2005 infant feeding survey (IFS) and women delivering at Royal Hampshire County Hospital, Winchester (data from routine hospital data, 2007) (RHCH)

The strengths of this analysis include a large cohort of 1140 infants at general risk of developing food hypersensitivity with good follow-up to 24 months and a diagnosis using the gold standard methodology of a DBPCFC. Additionally, the methodology allowed for the prospective identification and clinical assessment of infants reacting to foods which reduced the likelihood of tolerance developing before a diagnosis could be confirmed. The prospective design also reduced the likelihood of recall bias during data collection and the prospective collection of food diary data is unique in a cohort of this size. Limitations of our analysis are that the study population is not fully representative of the population from which it was recruited since our mothers are older (Fig. 3). Also, the control infants were 5 months older than the cases as they were matched by birth but recruited after the cases were assessed; since control infants had never had any adverse reactions to food we do not expect this difference to impact on any of the factors included in the risk factor analyses. Additional limitations are that the numbers of infants diagnosed as reactive to foods were too small to enable all the likely risk factors to be detected, or to allow us to look at individual food reactions to determine whether different foods behave differently and have different risk factors associated with them. Also, despite the prospective nature of the study, reverse causality could not be completely avoided as clinical symptoms can lead to treatments which are subsequently associated with the condition in an analysis, as was the case for anti-reflux medication. Furthermore, a diagnosis of allergy can result in a change of behaviour as was the case for when mothers first introduced egg into the diet. However, where there was a suspicion of reverse causality, the variable was not included in the final multivariable analysis thus reducing its effect on the final study findings. Finally, the risk factor investigation represents an exploratory analysis and needs to be replicated, since possible regional differences within the UK may mean the findings from this study are not necessarily nationally representative.

## Conclusions

This study presents unique data from the UK on both IgE-mediated food allergy and non IgE-mediated food hypersensitivity in early childhood. It found the cumulative incidence of DBPCFC confirmed food hypersensitivity to be 5.0 % (3.7–6.7). Our results also suggest that different factors may affect the development of IgE-mediated and non-IgE-mediated reactions to food in infants and young children. However, it is important that this exploratory analysis is confirmed in other cohorts. Analysis of the EuroPrevall data from all the birth cohort centres for all food reactions can build upon this work and also investigate any geographical differences to food hypersensitivity reactions.

## Additional file

**Additional file 1.** Additional tables of participant characteristics.

## Abbreviations

aOR: adjusted odds ratio
DBPCFC: double blind placebo controlled food challenge
PIFA Study: prevalence of infant food allergy study
SPT: skin prick test
SpIgE: specific immunoglobulin E
WTCRF: Wellcome Trust Clinical Research Facility
