# Supplementary material for: Incidence and risk factors for food hypersensitivity in UK infants: results from a birth cohort study
Source: Clin Transl Allergy. 2016 Jan 26;6:1. doi: 10.1186/s13601-016-0089-8 (PMC4727377; doi:10.1186/s13601-016-0089-8)
Supplement: Supplementary file 1 — Additional file 1. Additional tables of participant characteristics. [file 13601_2016_89_MOESM1_ESM.docx]

**Supplementary Files**

**Table S1** Maternal, Perinatal, Maternal nutrition during pregnancy and lactation and environmental factors

|  |  | **All participants (n=1140)** | **Participants with food hypersensitivity (n=41)** | **Control participants (n=82)** | **p-value** |
| --- | --- | --- | --- | --- | --- |

| ***Pregnancy*** | |  |  |  |  |
| --- | --- | --- | --- | --- | --- |
| Mean maternal pre-pregnancy weight, kg | | 65.6 (13.5) | 65.9 (14.0) | 64.0 (11.5) | 0.558 |
| Mean maternal pre-pregnancy height, cm | | 165.2 (6.8) | 164.1 (5.9) | 165.0 (6.6) | 0.690 |
| Median parity | | 0.0 (0.0-1.0 ) | 0.0 (0.0-1.0) | 0.0 (0.0-1.0) | 0.729 |
| Singleton pregnancy | | 1109 (97.7%) | 40 (97.6%) | 82 (100.0%) | 0.333 |
| Maternal smoking in pregnancy | | 70 (6.1%) | 1 (2.4%) | 3 (3.7%) | 1.000 |
| Other household smoking in pregnancy | | 196 (17.2%) | 8 (19.5%) | 5 (6.1%) | 0.027 |
| Aspirin/paracetamol during pregnancy | | 612 (68.8%) | 30 (81.1%) | 49 (69.0%) | 0.132 |
| Any anti-inflammatory during pregnancy | | 52 (5.8%) | 4 (11.1%) | 7 (9.9%) | 0.542 |
| Maternal antibiotics during pregnancy | | 177 (20%) | 9 (22.0%) | 19 (23.3%) | 0.286 |
| Mode of delivery | normal | 581 (51.3%) | 20 (51.3%) | 37 (45.7%) | 0.860 |
|  | caesarean | 348 (30.7%) | 13 (31.7%) | 20 (24.4%) |  |
|  | forceps | 106 (9.4%) | 3 (7.3%) | 12 (14.8%) |  |
| Mean gestation, weeks | | 39.4 (1.5) | 39.5 (1.7) | 40.0 (1.4) | 0.062 |
| Mean birth weight, grams | | 3459 (523) | 3480 (470) | 3370 (562) | 0.913 |
| Antibiotics in first week of age | | 5 (0.4%) | 0 (0.0%) | 0 (0.0%) | 1.000 |

| ***Diet during Pregnancy:*** | | | | | | |
| --- | --- | --- | --- | --- | --- | --- |
| Egg/egg products | Did not consume | 66 (7.4%) | | 2 (4.9%) | 3 (3.7%) | 0.561 |
|  | Reduced intake | 165 (18.4%) | | 10 (24.4%) | 13 (15.9%) | 0.579 |
| Milk/milk products | Did not consume | 10 (1.1%) | | 2 (4.9%) | 1 (1.2%) | 0.274 |
|  | Reduced intake | 35 (3.9%) | | 1 (2.4%) | 1 (1.3%) | 0.777 |
| Soy/soy products | Did not consume | 692 (77.4%) | | 28 (68.3%) | 52 (63.4%) | 0.488 |
|  | Reduced intake | 11 (1.2%) | | 0 (0.0%) | 0 (0.0%) | 0.195 |
| Peanut/peanut products | Did not consume | 418 (47.0%) | | 19 (46.3%) | 28 (34.1%) | 0.147 |
|  | Reduced intake | 181 (20.3%) | | 8 (19.5%) | 20 (24.4%) | 0.856 |
| Tree nuts/tree nut products | Did not consume | 282 (31.7%) | | 20 (48.8%) | 22 (26.8%) | 0.069 |
|  | Reduced intake | 161 (18.1%) | | 8 (19.5%) | 13 (15.9%) | 0.512 |
| Full maternal diet | | 39 (4.3%) | | 0 (0.0%) | 5 (6.1%) | 0.230 |
| Consume probiotics | | 269 (30.3%) | | 13 (31.7%) | 16 (19.5%) | 0.120 |
| Took folic acid supplements | | 777 (88.1%) | | 36 (87.8%) | 69 (84.1%) | 0.345 |
| Took multi vitamins | | 489 (55.5%) | | 24 (58.5%) | 47(57.3%) | 0.495 |
| Took vitamin D supplements | | 12 (1.4%) | | 2 (4.9%) | 1 (1.2%) | 0.345 |
| Took fish oil capsules | | 102 (11.6%) | | 3 (9.8%) | 13 (17.0%) | 0.235 |
| ***Diet during Breast feeding:*** | | | | | | |
| Ever breast fed | | | 653 (91.3%) | 38 (92.7%) | 79 (96.3%) | 0.210 |
| Egg/egg products | Did not consume | | 41 (6.3%) | 3 (7.9%) | 2 (2.5%) | 0.215 |
|  | Reduced intake | | 46 (7.0%) | 6 (15.8%) | 5 (6.3%) | 0.119 |
| Milk/milk products | Did not consume | | 10 (1.5%) | 2 (5.3%) | 1 (1.3%) | 0.266 |
|  | Reduced intake | | 18 (2.8%) | 4 (10.5%) | 1 (1.3%) | 0.048 |
| Soy/soy products | Did not consume | | 532 (81.5%) | 22 (57.9%) | 55 (69.6%) | 0.048 |
|  | Reduced intake | | 26 (4.0%) | 1 (2.6%) | 0 (0.0%) | 0.382 |
| Peanut/peanut products | Did not consume | | 343 (52.5%) | 18 (47.4%) | 36 (45.6%) | 0.526 |
|  | Reduced intake | | 342 (52.4%) | 15 (39.5%) | 26 (32.9%) | 0.451 |
| Tree nut/tree nut products | Did not consume | | 235 (36.0%) | 17 (44.7%) | 24 (30.4%) | 0.139 |
|  | Reduced intake | | 175 (26.8%) | 8 (21.1%) | 13 (16.5%) | 0.406 |
| Consumed probiotics | | | 178 (27.3%) | 11 (28.9%) | 16 (20.3%) | 0.277 |
| Took folic acid supplements | | | 85 (12.2%) | 8 (21.1%) | 15 (19.0%) | 0.729 |
| Took multi vitamins | | | 237 (33.7%) | 10 (26.3%) | 29 (36.7%) | 0.230 |
| Took vitamin D supplements | | | 7 (1.1%) | 2 (5.3%) | 1 (1.3%) | 0.257 |
| Took fish oil capsules | | | 51 (7.8%) | 2 (5.3%) | 5 (6.3%) | 0.571 |

| ***Environmental*** | |  |  |  |  |
| --- | --- | --- | --- | --- | --- |
| Urban living Environment | | 273 (23.9%) | 8 (19.5%) | 11(13.4%) | 0.601 |
| Live on a main road | | 84 (9.1%) | 3 (7.3%) | 5 (6.1%) | 0.535 |
| Cat at home | | 325 (28.5%) | 10 (24.4%) | 24 (29.3%) | 0.365 |
| Dog at home | | 204 (17.9%) | 13 (31.7%) | 12 (14.6%) | 0.026 |
| Mould in home | | 49 (13.2%) | 5 (12.2%) | 8 (9.8%) | 0.457 |
| Type of flooring where baby sleeps | Carpet | 970 (86.7%) | 37 (90.2%) | 72 (87.8%) | 0.757 |
|  | Wooden, laminate or parquet | 138 (12.1%) | 4 (9.8%) | 9 (11.0%) |  |
|  | Linoleum or vinyl tiles | 10 (1.0%) | 0 (0.0%) | 1 (1.2%) |  |
| Type of mattress your baby sleeps on | Foam | 765 (67.1%) | 29 (70.7%) | 51 (62.2%) | 0.362 |
|  | Synthetic | 148 (13.0%) | 10 (24.4%) | 22 (26.8%) |  |
|  | Other | 87 (7.6%) | 2 (4.9%) | 9 (11.0%) |  |
| Cleaning kitchen work surfaces | Non-bactericidal | 358 (31.4%) | 12 (29.3%) | 27 (32.9%) | 0.396 |
|  | Bactericidal | 658 (57.8%) | 27 (65.9%) | 50 (61.0%) |  |
|  | Neither | 108 (9.5%) | 1 (2.4%) | 5 (6.1%) |  |
|  | Don’t know | 8 (0.7%) | 1 (2.4%) | 0 (0.0%) |  |
| Cleaning table where you eat | Spray cleaner | 598 (52.5%) | 26 (63.4%) | 38 (46.3%) | 0.131 |
|  | Soap and Water | 282 (24.9%) | 10 (24.4%) | 19 (23.2%) |  |
|  | Just water | 77 (6.8%) | 2 (4.9%) | 6 (7.3%) |  |
|  | None of these | 176 (15.4%) | 3 (7.3%) | 19 (23.2%) |  |
| Pacifier/dummy | Latex | 81 (8.8%) | 5 (12.2%) | 8 (9.8%) | 0.202 |
|  | Silicon | 307 (33.4%) | 21 (51.2%) | 32 (39.0%) |  |
| Attendance at day care or a nursery | | 401 (44.8%) | 13 (31.7%) | 19 (23.2%) | 0.246 |
| Mean age when started day care or a nursery, months | | 8.5 (3.1) | 7.3 (2.19) | 8.74 (3.74) | 0.269 |

Figures are numbers (%) in each group, means (SD) or medians (25^th^, 75^th^ centiles) unless specified. P-values relate to a comparison between cases and control; they represent a chi squared test for categorical data, Mann Whitney U test for non-parametric data and two sample t test for parametric data. All data was not available for all participants.

**Table S2** Details of the causative foods for all infants with food hypersensitivity (DBPCFC positive) and also divided by food hypersensitivity phenotype

| Food | DBPCFC Confirmed food hypersensitivity (n=41) | IgE-food allergy (IgE-mediated food hypersensitivity) (n=21) | Non-IgE-mediated food hypersensitivity (n=20) |
| --- | --- | --- | --- |
| Cows’ milk | 20 | 6 | 14 |
| Hens’ Egg | 22 | 17 | 5 |
| Peanut | 6 | 5 | 1 |
| Soy | 3 | 1 | 2 |
| Wheat | 2 | 1 | 1 |
| Fish | 1 | 1 | 0 |
| Lentil | 1 | 0 | 1 |
| Broccoli | 1 | 0 | 1 |

All but one infant had SpIgE measured. The infant for whom there is no SpIgE data was SPT negative and was characterized as having non-IgE mediated food hypersensitivity and was reactive to milk.

**Table S3 Comparison of maternal, paternal, nutritional and environmental factors for participants with IgE mediated food allergy and Non- IgE food hypersensitivity**

|  | | | | Participants with IgE mediated food (n=21) | | | | Participants with non-IgE food hypersensitivity (n=20) | p- value |
| --- | --- | --- | --- | --- | --- | --- | --- | --- | --- |
| Median age of child, months | | | | 12.0 | | | | 7.2 | 0.555 |
| Presenting symptoms: | | | |  | | | |  |  |
| Gastrointestinal | | | | 3 (14.3%) | | | | 8 (40%) | 0.077 |
| Cutaneous (eczema and/or urticarial) | | | | 13 (61.9%)  (3 urticaria) | | | | 8 (40%)  (1 urticaria) | 0.342 |
| Respiratory | | | | 2 (9.5%) | | | | 3 (15%) | 0.548 |
| Other | | | | 2 (9.5%) | | | | 1 (5%) | 0.846 |
| None | | | | 1 (4.8%) | | | | 0 (0%) |  |
| How soon did symptoms appear, minutes (SE)* [Range] | | | | 16.8  (8.98) [0-120 | | | | 339.2  (247.15) [0-4320] |  |
| Positive specific IgE (≥0.35 kU/l) | | | | 21 | | | | 0 |  |
| Mean specific IgE, kU/l | | | | 5.07 | | | | 0 |  |
| Positive SPT (≥3 mm) | | | | 17 | | | | 0 |  |
| Median SPT weal diameter, mm | | | | 5.1 | | | | 0 |  |
| Current eczema | | | | 21 (100 5 0 | | | | 12 (60%) | 0.015 |
| Current wheeze/asthma | | | | 9 (42.9%) | | | | 8 (40%) | 0.795 |
| Current rhinitis | | | | 8 (38.1%) | | | | 6 (30%) | 0.637 |
| Ethnicity (n=1133) : Caucasian | | | | 20 | | | | 19 | 0.367 |
| non-caucasian | | | | 1 | | | | 1 |  |
| Age mother (n=1136) mean, years (SD) | | | | 33.1 | | | | 30.0 | 0.075 |
| Age father ( N=1136) mean, years (SD) | | | | 35.0 | | | | 31.3 | 0.048 |
| Highest education of parents: | | | |  | | | |  |  |
| Low (up to 12y) | | | | 3 | | | | 4 | 0.367 |
| Intermediate (>12y, e.g. college) | | | | 5 | | | | 8 |  |
| High (e.g. university) | | | | 13 | | | | 8 |  |
|  | | | |  | | | |  |  |
| Allergies in family | | | |  | | | |  |  |
| Mother atopic (A, AR or E) | | | | 20 | | | | 16 | 0.292 |
| Father atopic (A, AR or E) | | | | 13 | | | | 10 | 0.749 |
| Mother food hypersensitivity | | | | 2 | | | | 9 | 0.013 |
| Father food hypersensitivity | | | | 4 | | | | 2 | 0.376 |
|  | | | |  | | | |  |  |
| Urban living environment | | | | 1 | | | | 7 | 0.015 |
| Number of sibs at home mean (SD) | | | | 0.62 | | | | 0.45 | 0.199 |
| Female sex (n=1140) | | | | 6 | | | | 11 | 0.279 |
| Season of birth (n=1139) | | | |  | | | |  |  |
| summer | | | | 8 | | | | 9 | 0.934 |
| autumn | | | | 3 | | | | 5 |  |
| winter | | | | 5 | | | | 3 |  |
| spring | | | | 5 | | | | 3 |  |
| Mean maternal pre-pregnancy weight, kg (n=979) | | | | | 68.74 | | | 63.0 | 0.496 |
| Mean maternal pre-pregnancy height, cm (n=986) | | | | | 164.11 | | | 164.05 | 0.718 |
| Median parity (n=1140) | | | | | 0.00 | | | 0.5 | 0.415 |
| Singleton pregnancy (n=1135) | | | | | 20 | | | 20 | 0.323 |
| Maternal smoking in pregnancy (n=1140) | | | | | 1 | | | 0 | 0.323 |
| Other household smoking (n=1140) | | | | | 3 | | | 5 | 0.098 |
| Aspirin/paracetamol during pregnancy (n=890) | | | | | 15 | | | 15 | 0.855 |
| Any anti-inflammatory during pregnancy (n=894) | | | | | 4 | | | 0 | 0.058 |
| Mode of delivery | | normal delivery | | | 31 | | | 15 | 0.604 |
| (n=1132) | | caesarean | | | 9 | | | 4 |  |
|  | | forceps | | | 2 | | | 1 |  |
| Maternal antibiotics during pregnancy (n=886) | | | | | 5 | | | 4 | 1.000 |
| Mean gestation (n=1139) , weeks | | | | | 39.19 | | | 39.05 | 0.659 |
| Mean birth weight (n=1131), grams | | | | | 3465.00 | | | 3382.89 | 1.000 |
| Antibiotics in first week of age (n=1115) | | | | | 0 | | | 0 |  |
| During pregnancy: | | | | |  | | |  |  |
| Egg/egg products (n=896) | Did not consume | | | | 1 | | | 1 | 0.906 |
|  | Reduced intake | | | | 4 | | | 6 | 0.558 |
| Milk/milk products  (n=894) | Did not consume | | | | 0 | | | 2 | 0.115 |
|  | Reduced intake | | | | 1 | | | 0 | 0.638 |
| Soy/soy products (n=894) | Did not consume | | | | 15 | | | 13 | 0.506 |
|  | Reduced intake | | | | 0 | | | 0 | 0.310 |
| Peanut/peanut products (n=890) | Did not consume | | | | 11 | | | 8 | 0.765 |
|  | Reduced intake | | | | 3 | | | 5 | 0.240 |
| Tree nus/tree nut products (n=889) | Did not consume | | | | 9 | | | 11 | 0.231 |
|  | Reduced intake | | | | 4 | | | 4 | 0.675 |
| Full maternal diet ( n-897) | | | | | 0 | | | 0 |  |
| Consume probiotics (n=887) | | | | | 6 | | | 7 | 0.393 |
| Took folic acid supplements (n=882) | | | | | 19 | | | 17 | 0.243 |
| Took multi vitamins (n=881) | | | | | 10 | | | 14 | 0.388 |
| Took vitamin D supplements (n=881) | | | | | 2 | | | 0 | 0.451 |
| Took fish oil supplement | | | | | 0 | | | 4 | 0.025 |
| Ever breast fed (n=918) | | | | | 11 | | | 13 | 0.853 |
| *While breast feeding:* | | | | |  | | |  |  |
| Egg/egg products (n= 707) | Did not consume | | | | 17 | | | 16 | 0.546 |
|  | Reduced intake | | | | 2 | | | 4 | 0.371 |
| Milk/milk products (n=712) | Did not consume | | | | 18 | | | 16 | 0.146 |
|  | Reduced intake | | | | 1 | | | 3 | 0.289 |
| Soy/soy products (n=712) | Did not consume | | | | 7 | | | 7 | 0.494 |
|  | Reduced intake | | | | 0 | | | 1 | 0.310 |
| Peanut/peanut products (n=710) | Did not consume | | | | 9 | | | 9 | 1.000 |
|  | Reduced intake | | | | 8 | | | 7 | 0.310 |
| Tree nut/tree nut products (n=710) | Did not consume | | | | 11 | | | 8 | 0.738 |
|  | Reduced intake | | | | 4 | | | 9 | 0.264 |
| Consumed probiotics (n=707) | | | | | 2 | | | 9 | 0.011 |
| Took folic acid supplements ( n=697) | | | | | 3 | | | 4 | 0.427 |
| Took multi vitamins (n=704) | | | | | 5 | | | 4 | 0.569 |
| Took vitamin D supplements (n=696) | | | | | 1 | | | 1 | 0.967 |
| Took fish oil capsules (n=688) | | | | | 1 | | | 1 | 0.967 |
| Median duration of any breastfeeding, weeks | | | | | 20.00  (4.0-32.0) | | | 13.0  (2.25-25.25) | 0.336 |
| Median exclusive breast feeding duration, weeks | | | | | 2.5  (0.0-6.25) | | | 2.0  (0.0-14.5) | 0.806 |
| Median age at first solid/semi-solid food, weeks | | | | | 20.5  (17.25-24.0) | | | 17  (16-20.75) | 0.157 |
| Median age at first cows milk formula feed, weeks | | | | | 3.5  (1.0-11.5) | | | 3  (1-8.25) | 0.815 |
| Median age at first cows milk in any form, weeks | | | | | 3.0  (1.0-8.5) | | | 3  (1-8) | 0.883 |
| Median age at any hens’ egg in any form, weeks | | | | | 52.0  (42-53) | | | 52  (35.25-52) | 0.928 |
| Median age at first wheat in any form, weeks | | | | | 29  (24.75-33) | | | 25  (19.5-27) | 0.295 |
| Median age at first peanut in any form, weeks | | | | | 52  (52.0-52.0) | | | 52  (52.0-52.0) | 0.623 |
| Duration of concurrent breastfeeding and any solid food, week | | | | | 0.0  (0-20.0) | | | 0  (0-5) | 0.411 |
| Duration of concurrent breastfeeding and cows milk in any form, week | | | | | 7  (0.0-23.0) | | | 5.5  (0-9.25) | 0.153 |
| Mean healthy eating dietary pattern score from principal component analysis(16) | | | | | -0.294  (0.19) | | | -0.472  (0.21) | 0.771 |
| Maternal smoking | | |  | | |  | 1 | 0 | 0.101 |
| Anyone else smoking inside home | | |  | | |  | 3 | 5 | 0.098 |
| Live on a main road | | |  | | |  | 1 | 2 | 0.520 |
| Cat at home | | |  | | |  | 4 | 6 | 0.929 |
| Dog at home | | |  | | |  | 6 | 7 | 0.265 |
| Mould in home | | |  | | |  | 2 | 3 | 0.592 |
| Type of flooring where baby sleeps (split into categories) | | |  | | |  |  |  |  |
| Carpet | | | | | |  | 19 | 18 | 0.959 |
| Wooden, laminate, parquet | | | | | |  | 2 | 2 |  |
| Linoleum or vinyl tiles | | | | | |  | 0 | 0 |  |
| Type of mattress your baby sleeps on (split into categories) | | |  | | |  |  |  |  |
| Foam | | | | | |  | 13 | 16 | 0.930 |
| Synthetic | | | | | |  | 6 | 3 |  |
| Other | | | | | |  | 1 | 10 |  |
| Cleaning kitchen work surfaces (split into categories) | | |  | | |  |  |  |  |
| Non-bactericidal | | | | | |  | 8 | 4 | 0.237 |
| Bactericidal | | | | | |  | 11 | 16 |  |
| Neither | | | | | |  | 1 | 0 |  |
| Don’t know | | | | | |  | 1 | 0 |  |
| Cleaning table where you eat (split into categories) | | |  | | |  |  |  |  |
| Spray cleaner | | | | | |  | 11 | 15 | 0.471 |
| Soap and water | | | | | |  | 7 | 3 |  |
| Just water | | | | | |  | 1 | 1 |  |
| None of these | | | | | |  | 2 | 1 |  |
| Pacifier/dummy (split into categories) | | |  | | |  |  |  |  |
| Latex | | | | | |  | 0 | 5 | 0.262 |
| Silicon | | | | | |  | 9 | 12 |  |
| Attendance at day care or a nursery | | |  | | |  | 9 | 4 | 0.025 |
| Upper respiratory infection | | | | | |  |  |  |  |
|  | | | None | | |  | 4 | 5 | 0.810 |
|  | | | Occasionally | | |  | 12 | 8 |  |
|  | | | Often | | |  | 4 | 5 |  |
| Lower respiratory infection | | | | | |  |  |  |  |
|  | | | None | | |  | 17 | 17 | 0.290 |
|  | | | Occasionally | | |  | 3 | 1 |  |
|  | | | Often | | |  | 0 | 0 |  |
| Wheeze with upper respiratory infection | | | | | |  |  |  |  |
|  | | | None | | |  | 11 | 13 | 0.153 |
|  | | | Occasionally | | |  | 6 | 5 |  |
|  | | | Often | | |  | 3 | 0 |  |
| Bronchiolitis (bronchitis) | | | | | |  |  |  |  |
|  | | | None | | |  | 18 | 18 | 0.146 |
|  | | | Occasionally | | |  | 2 | 0 |  |
|  | | | Often | | |  | 1 | 0 |  |
| Middle ear infection | | |  | | |  |  |  |  |
|  | | | None | | |  | 17 | 15 | 1.000 |
|  | | | Occasionally | | |  | 3 | 3 |  |
|  | | | Often | | |  | 0 | 0 |  |
| Gastrointestinal illness | | |  | | |  |  |  |  |
|  | | | None | | |  | 16 | 14 | 0.565 |
|  | | | Occasionally | | |  | 4 | 3 |  |
|  | | | Often | | |  | 0 | 1 |  |
| Median number of occasions antibiotics were taken in the last 12 months | | |  | | |  | 1.0 | 1.0 | 0.964 |
| Average age when first received antibiotics, months (SE) | | |  | | |  | 8.00 | 7.78 | 0.284 |
| Received aspirin | | |  | | |  | 1 | 0 | 0.311 |
| Received paracetamol | | |  | | |  | 20 | 18 | 0.632 |
| Received anti-inflammatories (e.g. Ibuprofen, Nurofen) | | |  | | |  | 15 | 14 | 0.703 |
| Received anti-reflux medication | | |  | | |  | 4 | 7 | 0.283 |
| Received any vaccinations | | |  | | |  | 20 | 17 | 0.311 |
| Received any skin creams, lotions or powders | | |  | | |  | 18 | 15 | 0.631 |
| Mean age when started day care or a nursery, months | | |  | | |  | 7.44 | 7.0 | 0.182 |

Figures are numbers (%), median (25^th^, 75^th^ centile) or mean (SD) in each group unless specified. P-values relate to a comparison between cases and control; they represent a chi squared test for categorical data, Mann Whitney U test for non-parametric data and two sample t test for parametric data. *SE represents Standard Error of the mean

**Table S4** Characteristics of participants with food hypersensitivity and their controls at initial assessment divided by food hypersensitivity phenotype

|  | **Participants with IgE mediated food allergy (n=21)** | **Control participants (n=82)** | **p-value** |  |  | **Participants with non-IgE food hypersensitivity (n=20)** | **Control participants (n=82)** | **p-value** |
| --- | --- | --- | --- | --- | --- | --- | --- | --- |
| Median age of child, months | 12.0 | 14.5 | 0.299 |  |  | 7.2 | 14.5 | 0.002 |
| Diagnostic criteria: positive DBPCFC | 21 |  |  |  |  | 20 |  |  |
| history of anaphylaxis | 0 |  |  |  |  | 0 |  |  |
| Presenting symptoms: |  |  |  |  |  |  |  |  |
| Gastrointestinal | 3 (14.3%) |  |  |  |  | 8 (40%) |  |  |
| Cutaneous (eczema and/or urticarial) | 13 (61.9%)  (3 urticaria) |  |  |  |  | 8 (40%)  (1 urticaria) |  |  |
| Respiratory | 2 (9.5%) |  |  |  |  | 3 (15%) |  |  |
| Other | 2 (9.5%) |  |  |  |  | 1 (5%) |  |  |
| None | 1 (4.8%) |  |  |  |  | 0 (0%) |  |  |
| How soon did symptoms appear, minutes (SE)* [Range] | 16.8  (8.98) [0-120] |  |  |  |  | 339.2  (247.15) [0-4320] |  |  |
| Positive specific IgE (≥0.35 kU/l) | 21 |  |  |  |  | 0 |  |  |
| Mean specific IgE, kU/l | 5.07 |  |  |  |  | 0 |  |  |
| Positive SPT (≥3 mm) | 17 |  |  |  |  | 0 |  |  |
| Median SPT weal diameter, mm | 5.1 |  |  |  |  | 0 |  |  |
| Current eczema | 21 (100%) | 45 (54.9%) | <0.001 |  |  | 12 (60%) | 45 (54.9%) | 0.604 |
| Current wheeze/asthma | 9 (42.9%) | 21 (25.6%) | 0.176 |  |  | 8 (40%) | 21 (25.6%) | 0.255 |
| Current rhinitis | 8 (38.1%) | 11 (13.4%) | 0.023 |  |  | 6 (30%) | 11 (13.4%) | 0.087 |

*SE represents Standard Error of the mean

**Table S5** Demographic, socioeconomic and familial factors of the study participants divided by food hypersensitivity phenotype

|  |  | **Participants with IgE mediated food allergy (n=21)** | **Control participants (n=82)** | **p-value** |  | **Participants with non-IgE food hypersensitivity (n=20)** | **Control participants (n=82)** | **p-value** |
| --- | --- | --- | --- | --- | --- | --- | --- | --- |
| Ethnicity (n=1133) : Caucasian | | 20 | 82 | 0.204 |  | 19 | 82 | 0.196 |
| non-caucasian | | 1 | 0 |  |  | 1 | 0 |  |
| Age mother (n=1136) mean, years (SD) | | 33.1 | 32.2 | 0.364 |  | 30.0 | 32.2 | 0.033 |
| Age father ( N=1136) mean, years (SD) | | 35.0 | 34.0 | 0.362 |  | 31.3 | 34.0 | 0.048 |
|  | |  |  |  |  |  |  |  |
| Highest education of parents: | |  |  |  |  |  |  |  |
| Low (up to 12y) | | 3 | 14 | 0.923 |  | 4 | 14 | 0.342 |
| Intermediate (>12y, e.g. college) | | 5 | 21 |  |  | 8 | 21 |  |
| High (e.g. university) | | 13 | 47 |  |  | 8 | 47 |  |
|  | |  |  |  |  |  |  |  |
| Allergies in family | |  |  |  |  |  |  |  |
| Mother atopic (A, AR or E) | | 20 | 52 | 0.003 |  | 16 | 52 | 0.106 |
| Father atopic (A, AR or E) | | 13 | 50 | 1.000 |  | 10 | 50 | 0.606 |
| Mother food hypersensitivity | | 2 | 20 | 0.231 |  | 9 | 20 | 0.055 |
| Father food hypersensitivity | | 4 | 9 | 0.459 |  | 2 | 9 | 1.000 |
|  | |  |  |  |  |  |  |  |
| Urban living environment | | 1 | 11 | 0.184 |  | 7 | 11 | 0.147 |
| Number of sibs at home mean (SD) | | 0.62 | 0.56 | 0.714 |  | 0.45 | 0.56 | 0.334 |
| Female sex (n=1140) | | 6 | 36 | 0.225 |  | 11 | 36 | 0.456 |
|  | |  |  |  |  |  |  |  |
| Season of birth (n=1139) | |  |  |  |  |  |  |  |
| summer | | 8 | 37 | 0.238 |  | 9 | 37 | 0.961 |
| autumn | | 3 | 13 |  |  | 5 | 13 |  |
| winter | | 5 | 14 |  |  | 3 | 14 |  |
| spring | | 5 | 18 |  |  | 3 | 18 |  |

**Table S6** Maternal, paternal and perinatal factors divided by food hypersensitivity phenotype

|  |  | **Participants with IgE mediated food allergy (n=21)** | **Control participants (n=82)** | **p-value** |  | **Participants with non-IgE food hypersensitivity (n=20)** | **Control participants (n=82)** | **p-value** |
| --- | --- | --- | --- | --- | --- | --- | --- | --- |
| Mean maternal pre-pregnancy weight, kg (n=979) | | 68.74 | 64.01 | 0.748 |  | 63.0 | 64.01 | 0.764 |
| Mean maternal pre-pregnancy height, cm (n=986) | | 164.11 | 164.96 | 0.223 |  | 164.05 | 164.96 | 0.761 |
| Median parity (n=1140) | | 0.00 | 0.00 | 0.733 |  | 0.5 | 0.00 | - |
| Singleton pregnancy (n=1135) | | 20 | 82 | 0.204 |  | 20 | 82 | - |
| Maternal smoking in pregnancy (n=1140) | | 1 | 2 | 0.499 |  | 0 | 2 | 1.000 |
| Other household smoking (n=1140) | | 3 | 5 | 0.355 |  | 5 | 5 | 0.023 |
| Aspirin/paracetamol during pregnancy (n=890) | | 15 | 49 | 0.570 |  | 15 | 49 | 0.378 |
| Any anti-inflammatory during pregnancy (n=894) | | 4 | 7 | 0.235 |  | 0 | 7 | 0.338 |
| Mode of delivery normal delivery | | 31 | 44 | 0.846 |  | 15 | 44 | 0.583 |
| (n=1132) caesarean | | 9 | 26 |  |  | 4 | 26 |  |
| forceps | | 2 | 12 |  |  | 1 | 12 |  |
| Maternal antibiotics during pregnancy (n=886) | | 5 | 13 | 0.520 |  | 4 | 13 | 0.733 |
| Mean gestation (n=1139) , weeks | | 39.19 | 39.76 | 0.178 |  | 39.05 | 39.76 | 0.156 |
| Mean birth weight (n=1131), grams | | 3465.00 | 3533.52 | 0.990 |  | 3382.89 | 3533.52 | 0.496 |
| Antibiotics in first week of age (n=1115) | | 0 | 0 | - |  | 0 | 0 | - |

**Table S7** Maternal nutritional factors during pregnancy and lactation divided by food hypersensitivity phenotype

|  |  | | **Participants with IgE mediated food allergy (n=21)** | **Control participants (n=82)** | **p-value** |  | **Participants with non-IgE food hypersensitivity (n=20)** | **Control participants (n=82)** | **p-value** |
| --- | --- | --- | --- | --- | --- | --- | --- | --- | --- |
| **During pregnancy:** | | |  |  |  |  |  |  |  |
| Egg/egg products (n=896) | | Did not consume | 1 | 3 | 1.000 |  | 1 | 3 | 1.000 |
|  |  | Reduced intake | 4 | 13 | 0.881 |  | 6 | 13 | 0.371 |
| Milk/milk products  (n=894) | | Did not consume | 0 | 1 | 1.000 |  | 2 | 1 | 0.107 |
|  |  | Reduced intake | 1 | 1 | 0.478 |  | 0 | 1 | 0.883 |
| Soy/soy products (n=894) | | Did not consume | 15 | 52 | 0.773 |  | 13 | 52 | 1.000 |
|  |  | Reduced intake | 0 | 0 | - |  | 0 | 0 | 0.452 |
| Peanut/peanut products (n=890) | | Did not consume | 11 | 28 | 0.201 |  | 8 | 28 | 0.785 |
|  |  | Reduced intake | 3 | 19 | 0.581 |  | 5 | 19 | 0.387 |
| Tree nus/tree nut products (n=889) | | Did not consume | 9 | 22 | 0.279 |  | 11 | 22 | 0.030 |
|  |  | Reduced intake | 4 | 12 | 0.412 |  | 4 | 12 | 0.743 |
| Full maternal diet ( n-897) | | | 0 | 2 | 1.000 |  | 0 | 2 | 1.000 |
| Consume probiotics (n=887) | | | 6 | 16 | 0.553 |  | 7 | 16 | 0.220 |
| Took folic acid supplements (n=882) | | | 19 | 63 | 0.333 |  | 17 | 63 | 1.000 |
| Took multi vitamins (n=881) | | | 10 | 47 | 0.279 |  | 14 | 47 | 0.567 |
| Took vitamin D supplements (n=881) | | | 2 | 0 | 0.045 |  | 0 | 0 | - |
| Took fish oil supplement | | | 0 | 9 | 0.194 |  | 4 | 9 | 0.458 |
|  | | |  |  |  |  |  |  |  |
| Ever breast fed (n=918) | | | 11 | 57 | 0.121 |  | 13 | 57 | 0.594 |
| ***While breast feeding:*** | | |  |  |  |  |  |  |  |
| Egg/egg products (n= 707) | | Did not consume | 17 | 68 | 0.501 |  | 16 | 68 | 0.184 |
|  |  | Reduced intake | 2 | 5 | 0.631 |  | 4 | 5 | 0.084 |
| Milk/milk products (n=712) | | Did not consume | 18 | 69 | 1.000 |  | 16 | 69 | 0.105 |
|  |  | Reduced intake | 1 | 1 | 0.373 |  | 3 | 1 | 0.027 |
| Soy/soy products (n=712) | | Did not consume | 7 | 15 | 0.139 |  | 7 | 15 | 0.139 |
|  |  | Reduced intake | 0 | 0 | - |  | 1 | 0 | 0.222 |
| Peanut/peanut products (n=710) | | Did not consume | 9 | 34 | 1.000 |  | 9 | 34 | 1.000 |
|  |  | Reduced intake | 8 | 26 | 0.789 |  | 7 | 26 | 1.000 |
| Tree nut/tree nut products (n=710) | | Did not consume | 11 | 46 | 0.785 |  | 8 | 46 | 0.112 |
|  |  | Reduced intake | 4 | 13 | 0.750 |  |  | 13 |  |
| Consumed probiotics (n=707) | | | 2 | 16 | 0.342 |  | 9 | 16 | 0.039 |
| Took folic acid supplements ( n=697) | | | 3 | 15 | 0.703 |  | 4 | 15 | 0.895 |
| Took multi vitamins (n=704) | | | 5 | 29 | 0.524 |  | 4 | 29 | 0.389 |
| Took vitamin D supplements (n=696) | | | 1 | 1 | 0.369 |  | 1 | 1 | 0.354 |
| Took fish oil capsules (n=688) | | | 1 | 5 | 1.000 |  | 1 | 5 | 1.000 |

**Table S8** Infant nutritional factors determined from prospective diet diary data divided by food hypersensitivity phenotype

|  |  | Participants with IgE mediated food allergy (n=21) | Control participants (n=82) | p-value |  | Participants with non-IgE food hypersensitivity (n=20) | Control participants (n=82) | p-value |
| --- | --- | --- | --- | --- | --- | --- | --- | --- |
| Median duration of any breastfeeding, weeks | | 20.00  (4.0-32.0) | 24.0  (7.0-31.0) | 0.936 |  | 13.0  (2.25-25.25) | 24.0  (7.0-31.0) | 0.201 |
| Median exclusive breast feeding duration, weeks | | 2.5  (0.0-6.25) | 8.5  (4.0-15.0) | 0.816 |  | 2.0  (0.0-14.5) | 8.5  (4.0-15.0) | 0.878 |
| Median age at first solid/semi-solid food, weeks | | 20.5  (17.25-24.0) | 20  (17.0-23.0) | 0.976 |  | 17  (16-20.75) | 20  (17.0-23.0) | 0.002 |
| Median age at first cows milk formula feed, weeks | | 3.5  (1.0-11.5) | 5.0  (1.0-14.0) | 0.935 |  | 3  (1-8.25) | 5.0  (1.0-14.0) | 0.944 |
| Median age at first cows milk in any form, weeks | | 3.00  (1.0-8.5) | 5  (1.0-14.3) | 0.673 |  | 3  (1-8) | 5  (1.0-14.3) | 0.866 |
| Median age at any hens’ egg in any form, weeks | | 52.0  (42-53) | 35  (29.0-39.0) | 0.026 |  | 52  (35.25-52) | 35  (29.0-39.0) | 0.131 |
| Median age at first wheat in any form, weeks | | 29  (24.75-33) | 26  (25.0-29.0) | 0.074 |  | 25  19.5-27) | 26  (25.0-29.0) | 0.197 |
| Median age at first peanut in any form, weeks | | 52  (52.0-52.0) | 52  (52.0-52.0) | 0.738 |  | 52  (52.0-52.0) | 52  (52.0-52.0) | 0.744 |
| Duration of concurrent breastfeeding and any solid food, week | | 0.00  (0-20.0) | 0  (0.0-9.75) | 0.811 |  | 0  (0-5) | 0  (0.0-9.75) | 0.274 |
| Duration of concurrent breastfeeding and cows milk in any form, week | | 7  (0.0-23.0) | 9.0  (3.5-21.5) | 0.519 |  | 5.5  (0-9.25) | 9.0  (3.5-21.5) | 0.047 |
| Mean healthy eating dietary pattern score from principal component analysis(16) | | -0.294  (0.19) | 0.185  (0.11) | 0.001 |  | -0.472  (0.21) | 0.185  (0.11) | <0.001 |

Figures are median (25^th^, 75^th^ centiles) or mean (SD) in each group. p-values relate to a comparison between cases and control; they represent a Wilcoxon sum ranked test for non-parametric data and two sample t test for parametric data.

**Table S9** Environmental, health and medicinal factors divided by food hypersensitivity phenotype

|  |  | **Participants with IgE mediated food allergy (n=21)** | **Control participants (n=82)** | **p-value** |  | **Participants with food non-IgE hypersensitivity (n=20)** | **Control participants (n=82)** | **p-value** |
| --- | --- | --- | --- | --- | --- | --- | --- | --- |
|  |  |  |  |  |  |  |  |  |
| Maternal smoking |  | 1 | 2 | 0.499 |  | 0 | 2 | 1.000 |
| Anyone else smoking inside home |  | 3 | 5 | 0.355 |  | 5 | 5 | 0.023 |
| Live on a main road |  | 1 | 5 | 1.000 |  | 2 | 5 | 0.621 |
| Cat at home |  | 4 | 22 | 0.580 |  | 6 | 22 | 0.784 |
| Dog at home |  | 6 | 9 | 0.076 |  | 7 | 9 | 0.015 |
| Mould in home |  | 2 | 8 | 1.000 |  | 3 | 8 | 0.452 |
| Type of flooring where baby sleeps (split into categories) |  |  |  |  |  |  |  |  |
| Carpet |  | 19 | 72 | 0.860 |  | 18 | 72 | 0.875 |
| Wooden, laminate, parquet | | 2 | 9 |  |  | 2 | 9 |  |
| Linoleum or vinyl tiles |  | 0 | 1 |  |  | 0 | 1 |  |
| Type of mattress your baby sleeps on (split into categories) |  |  |  |  |  |  |  |  |
| Foam |  | 13 | 48 | 0.698 |  | 16 | 48 | 0.272 |
| Synthetic |  | 6 | 22 |  |  | 3 | 22 |  |
| Other |  | 1 | 9 |  |  | 10 | 9 |  |
| Cleaning kitchen work surfaces (split into categories) |  |  |  |  |  |  |  |  |
| Non-bactericidal |  | 8 | 27 | 0.231 |  | 4 | 27 | 0.223 |
| Bactericidal |  | 11 | 50 |  |  | 16 | 50 |  |
| Neither |  | 1 | 5 |  |  | 0 | 5 |  |
| Don’t know |  | 1 | 0 |  |  | 0 | 0 |  |
| Cleaning table where you eat (split into categories) |  |  |  |  |  |  |  |  |
| Spray cleaner |  | 11 | 38 | 0.475 |  | 15 | 38 | 0.118 |
| Soap and water |  | 7 | 19 |  |  | 3 | 19 |  |
| Just water |  | 1 | 6 |  |  | 1 | 6 |  |
| None of these |  | 2 | 19 |  |  | 1 | 19 |  |
| Pacifier/dummy (split into categories) |  |  |  |  |  |  |  |  |
| Latex |  | 0 | 8 | 0.808 |  | 5 | 8 |  |
| Silicon |  | 9 | 32 |  |  | 12 | 32 |  |
| Attendance at day care or a nursery |  | 9 | 19 | 0.105 |  | 4 | 19 | 1.000 |
| Upper respiratory infection | |  |  |  |  |  |  |  |
|  | None | 4 | 21 | 0.599 |  | 5 | 21 | 0.965 |
|  | Occasionally | 12 | 36 |  |  | 8 | 36 |  |
|  | Often | 4 | 19 |  |  | 5 | 19 |  |
| Lower respiratory infection | |  |  |  |  |  |  |  |
|  | None | 17 | 71 | 0.295 |  | 17 | 71 | 0.887 |
|  | Occasionally | 3 | 4 |  |  | 1 | 4 |  |
|  | Often | 0 | 1 |  |  | 0 | 1 |  |
| Wheeze with upper respiratory infection | |  |  |  |  |  |  |  |
|  | None | 11 | 59 | 0.038 |  | 13 | 59 | 0.615 |
|  | Occasionally | 6 | 15 |  |  | 5 | 15 |  |
|  | Often | 3 | 2 |  |  | 0 | 2 |  |
| Bronchiolitis (bronchitis) | |  |  |  |  |  |  |  |
|  | None | 18 | 74 | 0.019 |  | 18 | 74 | 1.000 |
|  | Occasionally | 2 | 0 |  |  | 0 | 0 |  |
|  | Often | 1 | 1 |  |  | 0 | 1 |  |
| Middle ear infection |  |  |  |  |  |  |  |  |
|  | None | 17 | 70 | 0.389 |  | 15 | 70 | 0.366 |
|  | Occasionally | 3 | 6 |  |  | 3 | 6 |  |
|  | Often | 0 | 0 |  |  | 0 | 0 |  |
| Gastrointestinal illness |  |  |  |  |  |  |  |  |
|  | None | 16 | 69 | 0.233 |  | 14 | 69 | 0.072 |
|  | Occasionally | 4 | 7 |  |  | 3 | 7 |  |
|  | Often | 0 | 0 |  |  | 1 | 0 |  |
| Median number of occasions antibiotics were taken in the last 12 months |  | 1.0 | 0.0 | 0.388 |  | 1 | 0.0 | - |
| Average age when first received antibiotics, months (SE) |  | 8.00 | 7.00 | 0.741 |  | 7.78 | 7.00 | - |
| Received aspirin |  | 1 | 0 | 0.208 |  | 0 | 0 | - |
| Received paracetamol |  | 20 | 74 | 1.000 |  | 18 | 74 | 1.00 |
| Received anti-inflammatories (e.g. Ibuprofen, Nurofen) |  | 15 | 51 | 0.595 |  | 14 | 51 | 0.571 |
| Received anti-reflux medication |  | 4 | 10 | 0.481 |  | 7 | 10 | 0.018 |
| Received any vaccinations |  | 20 | 73 | 1.000 |  | 17 | 73 | 0.355 |
| Received any skin creams, lotions or powders |  | 18 | 55 | 0.145 |  | 15 | 55 | 0.546 |
| Mean age when started day care or a nursery, months |  | 7.44 | 8.74 | 0.410 |  | 7.0 | 8.74 | - |

Figures are numbers (%), median (25^th^, 75^th^ centile) or mean (SD) in each group unless specified. P-values relate to a comparison between cases and control; they represent a chi squared test for categorical data, Mann Whitney U test for non-parametric data and two sample t test for parametric data.
